# Mother and Child and Their Relation to Oral Hygiene

**Published:** 1911-06-15

**Authors:** W. A. White

**Affiliations:** Phelps, N. Y.


### MOTHER AND CHILD AND THEIR RELATION TO ORAL HYGIENE.

W. A. WHITE, D.D.S., PHELPS, N. Y.

The builder in constructing his building seeks out good material, and various kinds of material, to compose his structure. He selects for its base or foundation, stone, brick or other suitable material. And so on throughout the building. So the child must have the various elements which enter into the various parts of its structures and tissues of its body incorporated there. If the builder places poor material in one part of his building, he has a weakened structure. And so it is with the child, unless proper assimilation takes place, there is a weakness, and in order to produce proper structure, we must have thorough assimilation of the food, and thorough assimilation of the food requires thorough digestion; and in order to have thorough digestion, we must have good teeth, and thorough mastication of the food in the mouth.

Prof. Michael, of Germany, says that mastication consists of two different acts. First, the canine teeth, tear pieces of the food and cut it up, and second, the food in turn must be ground and ground by the molars, until, with proper insalvation by uncontaminated saliva, the food is carried into the stomach. Under such conditions we have thorough assimilation, which we cannot have without thorough mastication and digestion.

Now, when we take into consideration the fact that ninety-seven per cent. of the school children are in need of dental attention, with seventy-five per cent. of this number unable to have such attention, I ask this body of medical men, is there any greater avenue through which our efforts may be directed, which will yield greater results for the physical betterment of mankind, than by instructing the children in our schools the manifold blessings resulting from a thorough knowledge of, and how best to exercise this knowledge?

It cannot be truly said by physicians that the condition of the mouth and the absence of teeth have no bearing on the development of the nourishment of the child or other individual, neither can it be maintained that an individual can live just as well and with the same amount of vitality without insalivation of the food. It is evident, therefore, that insalivation must take place before we can realize a normal digestion and have our food valuable. Thoroughly masticated white bread; twenty-four per cent. of it is converted into soluble sugar in one minute, and thirty-nine per cent. of it in five minutes. Then, as this food passes into the stomach, the saliva-ferments are active for from ten to twenty-five minutes in the stomach, until they are neutralized by stomach acids. This proves the great importance of insalivation, especially in starch food. And on this point we must remember that the eye specialists admit that decayed teeth cause indigestion, and indigestion causes eye-strain.

How many of you gentlemen have seen a child sent to school with a sore face and an aching tooth, wholly unfit to accomplish the work set before it? The swelling soon passes off, and the mother and the child assume that the trouble has all passed away; but you gentlemen, and particularly those of the dental profession, know that the trouble has not gone, but an abscess is forming in the tooth. A fistula develops and the discharge vitiates the saliva of the mouth, and contaminates the food entering the stomach, and there the trouble begins. The assimilation which should take place there is interfered with by the unhealthy condition of the food lying in the child's stomach.

These things can be overcome only by a thorough knowledge of oral hygiene, and I believe no greater favor can be granted, or extended to the mothers and the children than for the Department of Health of the great State of New York to have in connection with its work, a Bureau of Oral Hygiene, and then every mother will bestow a blessing on the State

Department of Health, and by so doing it will withstand the ravages of storm, time and temper.

Now, in regard to this instruction of the mothers, I can do no better than to read to you the words of Daniel Webster, who said, when he addressed an assemblage of ladies:

"It is by the promulgation of sound morals in the community, and more essentially by the training and instruction of the young, that woman performs her part toward the preservation of a free government. It is generally admitted that public liberty and the perpetuity of a free constitution rest on the virtue and intelligence of the community which enjoys it. How is that virtue to be inspired, and how is that intelligence to be communicated? Bonaparte once asked Mme. de Stael in what manner he could best promote the happiness of France. Her reply was full of political wisdome. She said, 'Instruct the mothers of the French people.' Mothers are, indeed, the affectionate and effective teachers of the human race. The mother begins her process of training with the infant in her arms. It is she who directs, so to speak, its first mental and spiritual pulsations. She conducts it along the impressionable years of childhood and youth, and hopes to deliver it to the stern conflicts and tumultuous scenes of life, armed by those good principles which her child receives from maternal care and love.

"If we draw within the circle of our contemplation the mother of a civilized nation, what do we see? We behold so many artificers working not on frail and perishable matter, but on the immortal mind, molding and fashioning beings who are to exist forever. We applaud the artist whose skill and genius present the mimic upon the canvas; we admire and celebrate the sculpture who works out that same image in enduring marble; but how insignificant are these achievements, though the highest and fairest in all the departments of art, in comparison with the great vocation of human mothers. They work not upon the canvas that shall perish, or the marble that shall crumble into dust, but upon mind spirit, which is to last forever, and which is to bear, for good or evil, through its duration, the impress of a mother's plastic hand."

I believe that the future holds great possibilities for the dental profession, that its duty of preserving and promoting the public health will make it one of the greatest public forces, if we but use our best efforts to encourage and foster the teachings which we advocate. The influence of such work cannot die, but will go on and on for years to come, as a rich inheritance for those who follow.—*The Dental Dispensary Record.*

